# Self-Administered Questionnaire to Screen for Polycystic Ovarian Syndrome

**DOI:** 10.1089/whr.2020.0073

**Published:** 2020-12-16

**Authors:** Bronwyn S. Bedrick, Ashley M. Eskew, Jorge E. Chavarro, Emily S. Jungheim

**Affiliations:** ^1^ Department of Obstetrics and Gynecology, Washington University School of Medicine, St. Louis, Missouri, USA.; ^2^ Department of Gynecology and Obstetrics, Johns Hopkins University, Baltimore, Maryland, USA.; ^3^ Department of Obstetrics and Gynecology, Atrium Health, Charlotte, North Carolina, USA.; ^4^ Harvard T. H. Chan School of Public Health, Harvard University, Boston, Massachusetts, USA.; ^5^ Department of Obstetrics and Gynecology, Northwestern University Feinberg School of Medicine, Chicago, Illinois, USA.

**Keywords:** depilatory, hyperandrogenism, polycystic ovary syndrome

## Abstract

***Background:*** Polycystic ovary syndrome (PCOS) is a common yet underdiagnosed endocrinopathy with potentially serious sequelae. A screening questionnaire for PCOS can improve early identification and diagnosis.

***Objective:*** The purpose of this study was to test the utility of a self-administered questionnaire to help identify women at risk for PCOS.

***Study Design:*** We recruited women ages 18–50 with and without PCOS as defined by modified Rotterdam criteria to complete a self-administered survey of common PCOS signs and symptoms. The survey included questions regarding menstrual cycle characteristics and hyperandrogenism as measured by images from the Ferriman-Gallwey (FG) scoring system, and by report of depilatory practices.

***Results:*** Fifty-one women with PCOS and 50 women without PCOS participated in this study. Many study participants were current users of hormonal contraceptives making it difficult to discern menstrual cycle characteristics. Hirsutism, defined by a modification of the FG score of ≥3 from the upper lip and abdomen based on self-assessments, provided a sensitivity of 76% and specificity of 70%, whereas report of any depilatory practices provided a sensitivity of 71% and specificity of 74%. The combined sensitivity of these measures was 93% with a specificity of 52%. In multivariate logistic regression, women who used depilatory techniques had an adjusted odds ratio (aOR) of PCOS of 6.6 (95% confidence interval [CI] 2.5–17.3, *p* = 0.0002). Those with obesity had similar aOR of PCOS (aOR 6.7, 95% CI 2.5–17.9, *p* = 0.0001). Addition of other variables did not improve model fit and the net sensitivity and specificity of these two variables did not improve those of depilatory practices and hirsutism.

***Conclusions:*** Self-report of depilatory practices or hirsutism is sensitive for identifying women with PCOS. Given the prevalence of PCOS in reproductive-age women and the potentially serious health sequelae, it would be worthwhile to include questions about terminal hair growth and depilatory practices when providing general medical care to reproductive-age women to determine if further testing and screening for PCOS are indicated. This tool may also be helpful in populations where complete diagnostic evaluation may not be feasible.

## Introduction

Polycystic Ovarian Syndrome (PCOS) is the most common endocrinopathy affecting reproductive-age women, with a prevalence ranging from 6% to 20%.^1,2^ PCOS is likely underdiagnosed, due, in part, to nonuniform diagnostic criteria, health care provider unfamiliarity, and diversity of PCOS phenotypes.^3–5^ In addition, many women may not recognize that they have the condition and not seek appropriate evaluation and treatment. Moreover, all current diagnostic criteria require extensive diagnostic evaluation to exclude multiple conditions with overlapping presentation complicating both clinical diagnosis and identification of cases in large population-based research studies in which ruling out exclusion diagnoses may not be feasible.

Between 30% and 70% of women with PCOS are obese,^6,7^ and PCOS has been associated with type 2 diabetes,^8,9^ hypertension,^10^ cardiovascular disease,^9–11^ anxiety,^12,13^ depression,^13–15^ infertility,^16^ and endometrial cancer.^17^ PCOS is associated with obstetrical risks, including gestational diabetes and preeclampsia.^18–20^

Studies have also demonstrated increased risk for children born to mothers with PCOS, including prematurity,^18–20^ neonatal intensive care unit admissions,^19^ attention-deficit hyperactivity disorder, and autism spectrum disorder.^13^ However, a study by Dokras et al. showed that physicians are often unaware of these health risks and cannot identify the appropriate diagnostic criteria for PCOS. This is more prevalent for general gynecologists and those with fewer patients with PCOS.^21^ Indeed, a large retrospective study found that there was a lower prevalence of PCOS in primary care clinics than in community samples, suggesting underdiagnosis despite suggestive symptoms.^22^ Given the long-term sequelae associated with PCOS, it is important to optimize the accuracy and frequency of diagnosis through screening and referral to appropriate specialists.

To simplify clinical definitions of hirsutism, previous studies have examined the utility of using only specific portions of Ferriman-Gallwey (FG) index as predictors of hirsutism. In 2000, Knochenhauer et al. examined 695 hyperandrogenic women and found that a hair growth score ≥2 on the chin and lower abdomen was a highly sensitive predictor for hirsutism.^23^ Examining almost 2000 women, Cook et al. found that a hair growth score of the chin, lower abdomen, and upper abdomen ≥3 was able to accurately discriminate between hirsute and non-hirsute women at the same level as a modified FG score of >7.^24^ These simplifications of the FG index would render clinical evaluations less invasive. However, they also would allow for easier self-evaluation.

In addition, a questionnaire that identifies women who should undergo diagnostic workup for PCOS could reduce the number of women who go undiagnosed and untreated. Furthermore, a questionnaire for identifying PCOS would be useful in epidemiological studies of female reproductive health as such a questionnaire could be administered to large populations of women without requiring expensive visits and blood work to diagnose the condition.

The goal of this study is to utilize simplifications of the FG index to evaluate the efficacy of a self-administered questionnaire in distinguishing women at risk of PCOS.

## Methods

### Participants subjects

One hundred and one women 18–50 years of age were enrolled in St. Louis, MO, based on PCOS status. Fifty participants were recruited from the PCOS clinic at Washington University School of Medicine and had been previously diagnosed with PCOS, as defined by the modified Rotterdam criteria, including two of the following three features: clinical or biochemical signs of hyperandrogenism, polycystic appearing ovaries on ultrasound, and/or oligo-ovulation or anovulation.^25^ The remaining 51 participants were women who had never been diagnosed with PCOS and were recruited from the Washington University School of Medicine infertility clinic, local gynecology offices, and the community through a research registry.

For inclusion, confirmation of PCOS diagnosis was made through medical chart review. At the time of enrollment, women were informed that the purpose of the study was to help develop a screening questionnaire for PCOS. Exclusion criteria included inability to provide informed consent and non-English speakers. This study was approved by the Washington University Human Research Protection Office (IRB no. 201510026).

### Survey

Participants meeting study inclusion requirements were asked to complete a survey, which contained questions pertaining to the most common symptoms of PCOS. In addition, they were asked information about age, race, ethnicity, height, and weight if a current body mass index (BMI) was not available in the medical record.^10,26^ Survey responses were entered and stored on REDCap.^27^

#### Menstrual cycle characteristics

Women were asked to characterize their menstrual cycle length and regularity and use of contraceptive methods, including all forms of hormonal contraception or long-acting reversible contraceptives. Women using these forms of contraception (*n* = 53) and women who had been pregnant in the previous 6 months (*n* = 4) were excluded from analyses of cycle pattern.

#### Symptoms of hyperandrogenism

Women were asked about the presence of acne in the last 3 months. They were able to choose “no acne,” “1–4 pimples,” and “5 or more pimples” on cheeks, chin, and forehead. Acne severity was defined as “no acne,” “physiological acne,” or “clinical acne,” respectively, based on modified definitions from Poli et al.^28^

**FIG. 1.**
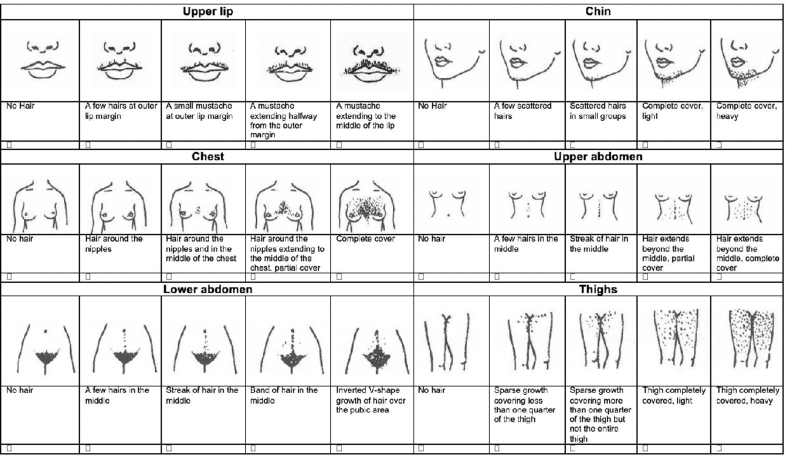
Ferriman-Gallwey images^39^ used for participant selection.

Women were asked whether they had ever used laser hair removal “in body parts other than bikini line, legs, or underarms.” They were also asked whether they had ever shaved, waxed, or bleached hair outside the previously mentioned areas. In addition, they were presented with images from the FG index to rate terminal hair growth in different body regions. Each region contained five images, the four original FG images as well as an FG image modified terminal hair to signify “no hair growth” (Fig. 1). Women selected the image corresponding to the extent of their terminal hair growth in the six body regions from the nine regions in the modified FG scale that are easiest to self-assess and most strongly associated with hirsutism: upper lip, chin, chest, upper and lower abdomen, and thighs.^23,29^ Hirsutism was defined by the simplified FG (sFG) score, or the sum of individual scores of the upper lip, lower abdomen, and upper abdomen, If the total sFG score was greater or equal to 3, the woman was considered to have hirsutism.^24^

### Statistical analysis

Chi-square, Fisher's exact, and Wilcoxon rank-sum tests were used to compare cases and controls, when appropriate. The sensitivity and specificity for sFG, individual body regions,^23^ and depilatory practices in identifying PCOS were calculated. To evaluate the use of multiple screening questions concurrently, net sensitivity and specificity were calculated for hirsutism and use of depilatory practices. Net sensitivity and specificity were calculated for the combined body regions of the lower abdomen and chin as described in Knochenhauer et al.^23^ To calculate the positive and negative predictive values of questions in the questionnaire, prevalence of hirsutism in PCOS was defined as 70%^30^ and prevalence of PCOS in the population was defined as 5%–15%.^5^

To evaluate the merit of including multiple variables in screening, we performed a stepwise multiple logistic regression, including the following characteristics: obesity, as defined by BMI ≥30 kg/m^2^, use of depilatory practices, hirsutism as defined by sFG, and presence of clinical acne.

For all analyses, *p* < 0.05 was considered statistically significant. SAS Version 9.4 (SAS Institute, Inc., Cary, NC) was used for all analyses.

## Results

Fifty-one women with PCOS and 50 women without PCOS were enrolled in the study. There were no significant differences in age or contraceptive use between groups (Table 1). The majority of women with PCOS identified as white, whereas women without PCOS were more heterogeneous; however, this difference was not statistically significant. Women with PCOS had a median BMI of 32 kg/m^2^, and 67% were obese. The median average BMI for women without PCOS was significantly lower at 25 kg/m^2^ (*p* < 0.0001), and only 22% were obese.

**Table 1.** Demographic and clinical characteristics by polycystic ovary syndrome diagnosis

Characteristic	PCOS (51)	No PCOS (50)	Unadjusted p-value
Age	28 (25–33)	28.5 (23–35)	0.83
BMI	32 (28–39)	25 (22–30)	<0.0001
Race			
White	50 (98)	40 (87)^a^	0.08
African American	1 (2)	4 (9)	
Other	0 (0)	2 (4)	
Menstrual pattern irregularity^b^	11 (55)	2 (10)	0.003
Contraception	26 (50)	26 (50)	0.92
COC	19 (37)	12 (24)	0.15
Depilatory practice			
Electrolysis	8 (16)	5 (10)	0.37
Shave, wax, bleach	38 (75)	14 (28)	<0.0001
Clinical acne^c^	19 (37)	10 (20)	0.06
Hair			
Total sFG	4 (3–6)	1 (0–3)	<0.0001
Hirsutism by sFG	37 (76)	14 (28)	<0.0001
Locations			
Chin	1 (1–2)	0 (0–1)	<0.0001
Upper lip	2 (1–3)	1 (0–1)	<0.0001
Chest	0 (0–1)	0 (0–1)	0.12
Upper abdomen	0 (0–1)	0 (0–1)	0.16
Lower abdomen	1 (2–3)	1 (0–1)	<0.0001
Thigh	2 (1–3)	1 (0–2)	0.009

^a^ Four participants had unknown race.

^b^ For patients not using hormonal contraception or intrauterine devices.

^c^ Clinical acne defined as 5+ pustules on chin, cheeks, and forehead within previous 3 months.

BMI, body mass index; COCs, combined oral contraceptive pills; PCOS, polycystic ovary syndrome; sFG, simplified Ferriman-Gallwey Categorical data represented as *n* (%), and continuous and integer variables represented as median (interquartile range).

Women with PCOS were more likely to have clinical acne than women without PCOS and were more likely to use depilatory techniques to remove terminal hair growth. When examining only women who were not using hormonal contraception (*n* = 24 PCOS and 24 non-PCOS women), women with PCOS were more likely to have menstrual irregularity (55% vs. 10%; *p* = 0.003). The majority (73%) of women with PCOS met criteria for hirsutism by sFG ≥3. For women with PCOS, 89% of those using combined oral contraceptives (COCs) and 63% not using oral contraceptives (OCPs) met criteria for hirsutism, but this difference was not statistically significant.

While a high percentage (28%) of non-PCOS women met criteria for hirsutism, the average total sFG was significantly higher in women with PCOS than in controls (*p* < 0.0001). Women with PCOS had significantly higher FG scores for terminal hair growth based on the modified FG images than non-PCOS women for all regions, except chest and upper abdomen. However, the median score for these regions for both cases and controls was low (Table 1).

The sensitivity and specificity of using sFG ≥3 for identifying PCOS were 76% and 70%, respectively (Table 2). Participating in any depilatory practice had similar sensitivities and specificities. When these two screening questions were combined, the net sensitivity was high at 93%, but specificity decreased to 52%. For regional terminal hair score of ≥1, sensitivities ranged from 47% to 92% and specificities from 36% to 68% (Table 2). As expected, as score cutoffs increased, sensitivities decreased and specificities increased (≥3 and ≥4, not shown). Net sensitivity and specificity for the lower abdomen and chin scores of ≥1 were 98% and 24%, respectively. However, increasing the score to ≥2 increased specificity to 74%, whereas sensitivity only decreased to 79%.

**Table 2.** Sensitivity and specificity for polycystic ovary syndrome diagnosis based on regional terminal hair growth and depilatory practices

	Sensitivity (%)	Specificity (%)
Hirsutism^a^	76	70
Depilatory practices^b^	71	74
Hirsutism and depilatory practices combined^c^	93	52
Obesity	76	70
Obesity and depilatory practices combined^d^	93	52
Chin		
≥1	78	58
≥2	47	92
Lower abdomen		
≥1	92	41
≥2	61	80
Combined regions^e^		
≥1	98	24
≥2	79	74
Upper lip		
≥1	88	36
≥2	53	78
Upper abdomen		
≥1	47	69
≥2	22	83
Chest		
≥1	48	64
≥2	18	94
Thighs		
≥1	88	36
≥2	53	66

^a^ Hirsutism is defined as sFG ≥3.

^b^ Depilatory practices include shaving, waxing, or bleaching hair, or use of electrolysis on the face, chest, or abdomen.

^c^ Net sensitivity and specificity for hirsutism or depilatory practices.

^d^ Net sensitivity and specificity for obesity and depilatory practices.

^e^ Net sensitivity and specificity for combined body regions lower abdomen and chin.

sFG, simplified Ferriman-Gallwey index.

Positive predictive value (PPV) for identifying PCOS using sFG ≥3 ranged from 0.11 to 0.31 (Table 3). PPV ranges were similar for depilatory practices. The PPV for parallel use of both questions was higher at 0.14–0.36. Negative predictive values for these metrics were significantly higher.

**Table 3.** Positive and negative predictive values for hirsutism, depilatory practices, and combined lower abdomen and chin regions by varying polycystic ovary syndrome prevalence

Screening	Prevalence (%)	PPV	NPV
Hirsutism^a^	5	0.12	0.98
	10	0.22	0.96
	15	0.31	0.94
Depilatory practices^b^	5	0.11	0.98
	10	0.21	0.96
	15	0.29	0.94
Combined questions^c^	5	0.14	0.99
	10	0.26	0.99
	15	0.35	0.98
Lower abdomen and chin ≥1^d^	5	0.15	0.996
	10	0.27	0.99
	15	0.37	0.99
Lower abdomen and chin ≥2^e^	5	0.12	0.99
	10	0.23	0.97
	15	0.32	0.95

^a^ Hirsutism as defined by sFG.

^b^ Depilatory practices include shaving, waxing, or bleaching hair, or use of electrolysis on the face, chest, or abdomen.

^c^ For net sensitivity and specificity of hirsutism or depilatory practices.

^d^ For net sensitivity and specificity of lower abdomen and chin each ≥1.

^e^ For net sensitivity and specificity of lower abdomen and chin each ≥2.

NPV, negative predictive value; PPV, positive predictive value.

Together, obesity and using depilatory practices were significant predictors for PCOS diagnosis (Table 4). Inclusion of additional variables, such as hirsutism, presence of clinical acne, or menstrual irregularity in those not on contraceptives, did not improve the fit of the model significantly. *R*^2^ of the model was 0.41 and c-statistic was 0.81. Given that over 50% of women in our cohort were using contraception, analyses examining menstrual irregularity were not performed. Given the significance of these two predictors to the model, we also calculated the net sensitivity (93%) and specificity (52%) of obesity and the use of depilatory practices—the same as for depilatory practices and hirsutism by sFG. Therefore, the combinations of these questions did not improve upon the PPV and negative predictive value for hirsutism and depilatory practices.

**Table 4.** Multivariate logistic model for polycystic ovary syndrome prediction

Variable	aOR	95% CI	p
Obesity	6.7	2.5–17.9	0.0001
Depilatory practices	6.6	2.5–17.3	0.0002

aOR, adjusted odds ratio; CI, confidence interval.

## Discussion

### Principle findings

We found that asking women about their male-patterned hair growth and depilation practices through a self-administered questionnaire has high sensitivity and moderate specificity in predicting PCOS diagnosis, making questions regarding these practices ideal for PCOS screening.

### Results

Consistent with previous work, the prevalence of hirsutism in our cohort of women with PCOS was 73%,^30^ and obesity was a strong predictor of PCOS.^31,32^ Positive responses for hirsutism as defined by sFG or for depilatory practice had sensitivity and specificity of over 70%. Positive responses to both of these screening questions gave a high sensitivity of 93%, but lower specificity at 52%.

### Clinical implications

PCOS is associated with a myriad of poor health outcomes for women,^8–20^ as well as their offspring.^13,18–20^ In addition to poor health outcomes, women with PCOS have lower markers of quality of life, both physically and psychologically when compared to age-matched controls.^33^ It is estimated that PCOS costs the United States health care system $4.4 billion dollars throughout a woman's reproductive lifespan. However, only 2% of this cost is spent on initial evaluation.^34^ Furthermore, the diagnostic process for many women with PCOS is inefficient and unsatisfactory. Gibson-Helm et al. found that more than 2 years and two health professionals were needed for accurate diagnosis of PCOS in >33% of cases.^35^ In a large Australian cohort of women with PCOS, quality of life was associated with perceived quality of information given about their diagnosis.^33^ Gaps in physician knowledge about the risks of PCOS and delays in diagnosis result in missed recommended screenings, such as blood pressure, cholesterol, and hemoglobin A1C.^36^ Given the significant delay in diagnosis and impact on quality of life and long-term health, identification of women at risk of PCOS is vital.

The FG scoring system for hirsutism currently relies on physician evaluation. However, these examinations are cumbersome and frequently prohibitive in epidemiological studies. In addition, when women use depilatory techniques to remove hair, accurate evaluation by a physician may not be possible. Compared to more objective measurements of hirsutism, such as photographic scoring or hair measurements, scoring by the FG method is more subjective and has been shown to have high interobserver and intraobserver reliability.^37,38^

In 2005, Wild et al. asked 21 women with PCOS to score themselves and be scored by three trained professionals. They found considerable variability in scoring and concluded that self-scoring was not clinically useful. However, all scores were significantly higher than 6, indicating that all observers were in agreement that the women met criteria for hirsutism.^37^

More recently, Pedersen et al. sought to validate a questionnaire for use in the diagnosis of PCOS and noted that a history of infrequent menses, hirsutism, obesity, and acne was strongly predictive of a diagnosis of PCOS and developed a four-item questionnaire that yielded a sensitivity of 77% and specificity of 94%. However, in contrast to our study presented here, they recruited women with menstrual irregularity, hirsutism, and infertility, which limit generalizability. They also used the National Institutes of Health (NIH) diagnostic criteria for PCOS, which requires oligo-ovulation or anovulation and clinical or biochemical hyperandrogenism for diagnosis, which differs from our study.^39^

While self-evaluation may be less accurate than evaluation by a trained professional, this study demonstrates that women can accurately determine whether they have hirsutism. As a solution for high variability in scoring, Cook et al. introduced the simplified FG method, which reduces the number of body regions evaluated, while still accurately diagnosing hirsutism.^24^ In our study, we used the sFG to designate hirsutism; however, we asked patients to evaluate themselves. Given that 37% of generalists are unaware of the diagnostic criteria for PCOS,^21^ self-screening questionnaires may be able to improve targeted referrals to specialists.

### Research implications

Simple, self-administered questionnaires improve the ability to conduct large epidemiological studies, as they do not rely on expert evaluation. Implementation of a questionnaire to screen for PCOS, which includes questions about depilatory practices and hirsutism, may help in epidemiologic studies of PCOS.

### Strengths and limitations

We note three key strengths of this study. This adds to the extremely limited literature that has examined the utility of a screening questionnaire to identify women at risk for PCOS. As a chronic disease with significant long-term sequelae and lifestyle and medical interventions, PCOS is a prime disease for a screening questionnaire. This screening test is both sensitive and specific and has a high negative predictive value. While the PPV was relatively low, this reflects the overall prevalence of PCOS. Finally, the questions do not rely on menstrual irregularity. Given the large proportion of reproductive-age women who use contraception and whose menstrual cycles may be normalized or affected by these methods, it is vital that a screening tool does not rely on menstrual regularity.

Several limitations of our study must be considered. First, as with all case–control studies, recall and selection bias are two major considerations. For example, women with PCOS who report to clinics may be more symptomatic than women with PCOS who do not present for evaluation. Therefore, the women with PCOS in our study may be more symptomatic than undiagnosed PCOS patients. In addition, as the participants were not blinded to the purpose of the study, their responses are subject to recall bias.

Second, our cohort predominantly identified as white, which limits its generalizability. Given that terminal hair growth can vary by race and ethnicity, it is important for future studies to include a more heterogeneous sample. Depilatory techniques are frequently employed for removal of eyebrow hair in women without hirsutism. Our questionnaire did not specifically ask women if their facial hair removal was in areas with male-patterned growth, which may have led to falsely elevated proportions of women answering yes to this question.

Third, for several participants, self-reported weight and height were used to calculate BMI. While self-reported weight and height are not as precise as measurements conducted in clinic, they have been shown to be accurate.^26^

Fourth, given the large portion of women on OCPs, questions about menstrual cycle characteristics could not be used in the final model. Studies have remedied this problem by excluding women on COCs. However, exclusion of women on COCs biases results since symptomatic women are more likely to have been prescribed COCs. Also, given that ∼40% of reproductive-age women use some form of hormonal contraception or long-acting reversible contraceptive,^40^ it is important for a screening tool to identify individuals at risk of PCOS without relying on questions about menstrual regularity. Furthermore, asking women to recall their menstrual cycle characteristics before initiating hormonal contraception is not without limitations, including recall bias, which will increase the longer a woman is on contraception. Furthermore, cycle characteristics several years prior are not necessarily a good predictor of current cycle characteristics. Therefore, while it would be ideal to know current cycle characteristics of all women, this has limited utility in a general population.

## Conclusions

Straightforward self-screening questions on obesity, depilatory practices, and male-patterned terminal hair growth can aid in identifying women at risk for PCOS. This has significant implications for helping women and physicians correctly identify who should be further worked up for this endocrinopathy.

## Condensation

A questionnaire capturing self-report of depilatory practices and/or hirsutism is sensitive for identifying women with PCOS.

## Abbreviations Used

aOR: adjusted odds ratio
BMI: body mass index
CI: confidence interval
COC: combined oral contraceptive pill
NIH: National Institutes of Health
NPV: negative predictive value
OCP: oral contraceptive
PCOS: polycystic ovary syndrome
PPV: positive predictive value
sFG: simplified Ferriman-Gallwey

## References

[1] Knochenhauer ES, Key TJ, Kahsar-Miller M, Waggoner W, Boots LR, Azziz R Prevalence of the polycystic ovary syndrome in unselected black and white women of the southeastern United States: A prospective study. J Clin Endocrinol Metab 1998;83:3078–3082 9745406 10.1210/jcem.83.9.5090

[2] Bozdag G, Mumusoglu S, Zengin D, Karabulut E, Yildiz BO The prevalence and phenotypic features of polycystic ovary syndrome: A systematic review and meta-analysis. Hum Reprod 2016;31:2841–2855 27664216 10.1093/humrep/dew218

[3] Wijeyaratne CN, Dilini Udayangani SA, Balen AH Ethnic-specific polycystic ovary syndrome: Epidemiology, significance and implications. Expert Rev Endocrinol Metab 2013;8:71–79 30731654 10.1586/eem.12.73

[4] March WA, Moore VM, Willson KJ, Phillips DI, Norman RJ, Davies MJ The prevalence of polycystic ovary syndrome in a community sample assessed under contrasting diagnostic criteria. Hum Reprod 2010;25:544–551 19910321 10.1093/humrep/dep399

[5] Azziz R. Polycystic ovary syndrome. Obstet Gynecol 2018;132:321–336 29995717 10.1097/AOG.0000000000002698

[6] Vrbikova J, Hainer V Obesity and polycystic ovary syndrome. Obes Facts 2009;2:26–35 20054201 10.1159/000194971 PMC6444522

[7] Lim SS, Davies MJ, Norman RJ, Moran LJ Overweight, obesity and central obesity in women with polycystic ovary syndrome: A systematic review and meta-analysis. Hum Reprod Update 2012;18:618–637 22767467 10.1093/humupd/dms030

[8] Morgan CL, Jenkins-Jones S, Currie CJ, Rees DA Evaluation of adverse outcome in young women with polycystic ovary syndrome versus matched, reference controls: A retrospective, observational study. J Clin Endocrinol Metab 2012;97:3251–3260 22767635 10.1210/jc.2012-1690

[9] Mani H, Levy MJ, Davies MJ, et al. Diabetes and cardiovascular events in women with polycystic ovary syndrome: A 20-year retrospective cohort study. Clin Endocrinol (Oxf) 2013;78:926–934 23046078 10.1111/cen.12068

[10] Ollila ME, Kaikkonen K, Jarvelin MR, et al. Self-reported polycystic ovary syndrome is associated with hypertension: A Northern Finland birth cohort 1966 study. J Clin Endocrinol Metab 2019;104:1221–1231 30445634 10.1210/jc.2018-00570 PMC7296204

[11] de Groot PC, Dekkers OM, Romijn JA, Dieben SW, Helmerhorst FM PCOS, coronary heart disease, stroke and the influence of obesity: A systematic review and meta-analysis. Hum Reprod Update 2011;17:495–500 21335359 10.1093/humupd/dmr001

[12] Dokras A, Clifton S, Futterweit W, Wild R Increased prevalence of anxiety symptoms in women with polycystic ovary syndrome: Systematic review and meta-analysis. Fertil Steril 2012;97:225.e222–230.e222. 22127370 10.1016/j.fertnstert.2011.10.022

[13] Berni TR, Morgan CL, Berni ER, Rees DA Polycystic ovary syndrome is associated with adverse mental health and neurodevelopmental outcomes. J Clin Endocrinol Metab 2018;103:2116–2125 29648599 10.1210/jc.2017-02667

[14] Kerchner A, Lester W, Stuart SP, Dokras A Risk of depression and other mental health disorders in women with polycystic ovary syndrome: A longitudinal study. Fertil Steril 2009;91:207–212 18249398 10.1016/j.fertnstert.2007.11.022

[15] Dokras A, Clifton S, Futterweit W, Wild R Increased risk for abnormal depression scores in women with polycystic ovary syndrome: A systematic review and meta-analysis. Obstet Gynecol 2011;117:145–152 21173657 10.1097/AOG.0b013e318202b0a4

[16] ACOG practice bulletin no. 194: Polycystic ovary syndrome. Obstet Gynecol 2018;131:e157–e171 29794677 10.1097/AOG.0000000000002656

[17] Barry JA, Azizia MM, Hardiman PJ Risk of endometrial, ovarian and breast cancer in women with polycystic ovary syndrome: A systematic review and meta-analysis. Hum Reprod Update 2014;20:748–758 24688118 10.1093/humupd/dmu012 PMC4326303

[18] Roos N, Kieler H, Sahlin L, Ekman-Ordeberg G, Falconer H, Stephansson O Risk of adverse pregnancy outcomes in women with polycystic ovary syndrome: Population based cohort study. BMJ 2011;343:d6309 21998337 10.1136/bmj.d6309 PMC3192872

[19] Qin JZ, Pang LH, Li MJ, Fan XJ, Huang RD, Chen HY Obstetric complications in women with polycystic ovary syndrome: A systematic review and meta-analysis. Reprod Biol Endocrinol 2013;11:56 23800002 10.1186/1477-7827-11-56 PMC3737012

[20] Kjerulff LE, Sanchez-Ramos L, Duffy D Pregnancy outcomes in women with polycystic ovary syndrome: A metaanalysis. Am J Obstet Gynecol 2011;204:558.e551–558.e556. 21752757 10.1016/j.ajog.2011.03.021

[21] Dokras A, Saini S, Gibson-Helm M, Schulkin J, Cooney L, Teede H Gaps in knowledge among physicians regarding diagnostic criteria and management of polycystic ovary syndrome. Fertil Steril 2017;107:1380.e1381–1386.e1381. 28483503 10.1016/j.fertnstert.2017.04.011

[22] Ding T, Baio G, Hardiman PJ, Petersen I, Sammon C Diagnosis and management of polycystic ovary syndrome in the UK (2004–2014): A retrospective cohort study. BMJ Open 2016;6:e012461 10.1136/bmjopen-2016-012461 PMC4947736 27401369

[23] Knochenhauer ES, Hines G, Conway-Myers BA, Azziz R Examination of the chin or lower abdomen only for the prediction of hirsutism. Fertil Steril 2000;74:980–983 11056244 10.1016/s0015-0282(00)01602-2

[24] Cook H, Brennan K, Azziz R Reanalyzing the modified Ferriman-Gallwey score: Is there a simpler method for assessing the extent of hirsutism? Fertil Steril 2011;96:1266–1270. e1261. 21924716 10.1016/j.fertnstert.2011.08.022 PMC3205229

[25] Rotterdam: ESHRE ASRM-Sponsored PCOS Consensus Workshop Group. Revised 2003 consensus on diagnostic criteria and long-term health risks related to polycystic ovary syndrome. Fertil Steril 2004;81:19–25 10.1016/j.fertnstert.2003.10.004 14711538

[26] Rimm EB, Stampfer MJ, Colditz GA, Chute CG, Litin LB, Willett WC Validity of self-reported waist and hip circumferences in men and women. Epidemiology 1990;1:466–473 2090285 10.1097/00001648-199011000-00009

[27] Harris PA, Taylor R, Thielke R, Payne J, Gonzalez N, Conde JG Research electronic data capture (REDCap)—A metadata-driven methodology and workflow process for providing translational research informatics support. J Biomed Inform 2009;42:377–381 18929686 10.1016/j.jbi.2008.08.010 PMC2700030

[28] Poli F, Dreno B, Verschoore M. An epidemiological study of acne in female adults: Results of a survey conducted in France. J Eur Acad Dermatol Venerol 2001:541–545 10.1046/j.1468-3083.2001.00357.x 11843213

[29] Lunde O, Grottum P Body hair growth in women: Normal or hirsute. Am J Phys Anthropol 1984;64:307–313 6540999 10.1002/ajpa.1330640313

[30] Azziz R, Woods KS, Reyna R, Key TJ, Knochenhauer ES, Yildiz BO The prevalence and features of the polycystic ovary syndrome in an unselected population. J Clin Endocrinol Metab 2004;89:2745–2749 15181052 10.1210/jc.2003-032046

[31] Teede HJ, Joham AE, Paul E, et al. Longitudinal weight gain in women identified with polycystic ovary syndrome: Results of an observational study in young women. Obesity (Silver Spring) 2013;21:1526–1532 23818329 10.1002/oby.20213

[32] Alvarez-Blasco F, Botella-Carretero JI, San Millan JL, Escobar-Morreale HF Prevalence and characteristics of the polycystic ovary syndrome in overweight and obese women. Arch Intern Med 2006;166:2081–2086 17060537 10.1001/archinte.166.19.2081

[33] Ching HL, Burke V, Stuckey BG Quality of life and psychological morbidity in women with polycystic ovary syndrome: Body mass index, age and the provision of patient information are significant modifiers. Clin Endocrinol (Oxf) 2007;66:373–379 17302871 10.1111/j.1365-2265.2007.02742.x

[34] Azziz R, Marin C, Hoq L, Badamgarav E, Song P Health care-related economic burden of the polycystic ovary syndrome during the reproductive life span. J Clin Endocrinol Metab 2005;90:4650–4658 15944216 10.1210/jc.2005-0628

[35] Gibson-Helm M, Teede H, Dunaif A, Dokras A Delayed diagnosis and a lack of information associated with dissatisfaction in women with polycystic ovary syndrome. J Clin Endocrinol Metab 2017;102:604–612 27906550 10.1210/jc.2016-2963 PMC6283441

[36] Legro RS, Arslanian SA, Ehrmann DA, et al. Diagnosis and treatment of polycystic ovary syndrome: An Endocrine Society clinical practice guideline. J Clin Endocrinol Metab 2013;98:4565–4592 24151290 10.1210/jc.2013-2350 PMC5399492

[37] Wild RA, Vesely S, Beebe L, Whitsett T, Owen W Ferriman Gallwey self-scoring I: Performance assessment in women with polycystic ovary syndrome. J Clin Endocrinol Metab 2005;90:4112–4114 15827102 10.1210/jc.2004-2243

[38] Barth JH. How robust is the methodology for trials of therapy in hirsute women? Clin Endocrinol (Oxf) 1996;45:379–380 8959073 10.1046/j.1365-2265.1996.00828.x

[39] Pedersen SD, Brar S, Faris P, Corenblum B Polycystic ovary syndrome: Validated questionnaire for use in diagnosis. Can Fam Physician 2007;53:1042–1047. 1041. 17872783 PMC1949220

[40] Kavanaugh ML, Jerman J Contraceptive method use in the United States: Trends and characteristics between 2008, 2012 and 2014. Contraception 2018;97:14–21 29038071 10.1016/j.contraception.2017.10.003 PMC5959010

